# The Role of Mesenchymal Stem Cells in the Treatment of a Chronic Rhinosinusitis—An In Vivo Mouse Model

**DOI:** 10.3390/microorganisms9061182

**Published:** 2021-05-30

**Authors:** Veronica-Elena Trombitaș, Alina Anda Nagy, Cristian Berce, Emoke Pall, Flaviu Tăbăran, Aranka Ilea, Silviu Albu

**Affiliations:** 1 II-nd Department of Otolaryngology, Iuliu Hațieganu University of Medicine and Pharmacy, 400015 Cluj-Napoca, Romania; alinaandanagy@gmail.com (A.A.N.); silviualbu63@gmail.com (S.A.); 2 Department of Experimental Medicine, Iuliu Hațieganu University of Medicine and Pharmacy, 400000 Cluj-Napoca, Romania; cristi.berce@gmail.com; 3 Department of Reproduction, Obstetrics and Veterinary Gynecology, Faculty of Veterinary Medicine, University of Agricultural Science and Veterinary Medicine, 400372 Cluj-Napoca, Romania; pallemoke@gmail.com; 4 Department of Anatomic Pathology, Necropsy and Forensic Medicine, Faculty of Veterinary Medicine, University of Agricultural Science and Veterinary Medicine, 400372 Cluj-Napoca, Romania; flaviutabaran@gmail.com; 5 Department of Oral Rehabilitation, Oral Health and Dental Office Management, Iuliu Hațieganu University of Medicine and Pharmacy, 400000 Cluj-Napoca, Romania; aranka.ilea@umfcluj.ro

**Keywords:** mesenchymal stem cells, chronic rhinosinusitis, Aspergillus fumigatus, treatment, fluorescence microscopy, adipose tissue

## Abstract

**Objectives/Hypothesis:** It is acknowledged that the treatment of chronic rhinosinusitis (CRS) represents an important challenge for rhinology and for social and economic life. At present, one of the most common treatments for CRS is represented by local corticosteroids followed by endoscopic sinus surgery (ESS). Starting from the example of the mesenchymal stem cell’s (MSC) capacity to migrate and to modulate a real response in the nasal mucosa of an allergic rhinitis mouse model, we try to obtain a response in a CRS mouse model, using MSC derived by adipose tissue. The aim of this study is to demonstrate that the MSC can be used in CRS treatment and could change its priorities. **Methods:** Seventy female mice (6 MSC donor mice) were randomized in two stages of study, 32 Aspergillus fumigatus (Af) exposure mice (20 for histological comparison to 1st control mice and 12 for MSC administration, to CRS/MCS model) and 32 control mice (20 for histological comparison to CRS model and 12 for MSC administration and histological control to MSC model); in the first stage, the *Aspergillus fumigatus* (Af) CRS mouse model was targeted, in this section were included 64 (*n* = 32) mice (treated and control group). In order to assess the inflammation level (histological analysis), the animals were euthanized; in the second stage MSCs (1 × 10^6^/animal) were administered intravenously to a total of 24 (*n* = 24) mice (12 mice from the exposed group and 12 mice from the second control group). **Results:** After 12 weeks of Af intranasal instillation, the inflammation parameters evaluated indicated a severe diffuse chronic inflammation, associated with diffuse severe hyperplasia and mature diffuse squamous metaplasia. The MSCs’ injection via the ophthalmic vein induced important histopathological changes in the CRS experimental group, starting with the presence of MSCs in all samples and continuing with the important degenerative character of inflammation. **Conclusions:** MSC administration demonstrated a real improvement of CRS evolution on the CRS mouse model.

## 1. Introduction

Recently, researchers have defined different stem cell groups: hematopoietic stem cells (HSCs), mesenchymal stem cells (MSCs) and neural stem cells (NSCs). Mesenchymal stem cells (MSCs) represent a new direction of interest in regenerative medicine, thanks to their ability to differentiate into several cell types and to generate regenerative cytokines. MSCs are abundant in the bone marrow and fat of adults or fetuses; however, they can be isolated from muscles, placenta, dental pulp or umbilical cord blood [1,2]. In animal models, adipose tissue represents a good source of MSC, the sampling being done by surgical excision from the inguinal, abdominal or thoracic wall [2]. MSCs are able to differentiate into adipocytes, chondrocytes or osteocytes, and can repair and regenerate tissues, since they own the ability to identify the damaged organ and generate the cytokines with immunomodulation and immunosuppression characters [1,2]. The MSC culture has a very good plasticity because it can be easily collected and preserved for a period of time while also preserving its functionality. In the current literature, several studies demonstrated the regenerative ability of MSCs in animal models with induced injures, but we do not have data about the therapeutical properties of MSCs for naturally occurring diseases [2].

Lindsay et al. have created a CRS mouse model by exposing the nasal mucosa to an *Aspergillus fumigatus* (Af) extract for 12 weeks [3]. Using the same model, Tansavatdi et al. realized a pilot study to test the feasibility of mouse nasal wound healing and inflammatory response mediated by gene inflammatory expression in CRS [4]. Having chronic nasal inflammation as the central point, Kyu-Sup Cho et al. created an allergic rhinitis mouse model in 2009; they treated the nasal disease with allotransplanted i.v. stem cells and obtained an immunomodulatory effect characterized by the inhibition of chronic inflammation [5].

We aim to redirect the research to the noninvasive treatment of CRS, including the rebel evolution of CRS, especially the type with many recidives and exclusive surgery indication.

## 2. Materials and Methods

### 2.1. Study Design

This study is a prospective, randomized, partly-blinded, controlled trial.

#### 2.1.1. Animals and Housing

Seventy (*n* = 70) 4-week-old female Crl: CD1(ICR) mice weighing 21.15 ± 0.1487 g were used in the present study. The animals were housed in IVC cages (Tecniplast, Buguggiate, Italy) and had access to filtered sterile water and pelleted feed (Cantacuzino Institute, Romania) ad libitum. The bedding was standard wood chip bedding autoclaved before use (Lignocel, Rosenberg, Germany). The mice were kept in the Animal Facility of the “Iuliu Hațieganu” University of Medicine and Pharmacy in Cluj-Napoca, Romania at a standard temperature of 22 ± 2 °C, a relative humidity of 55 ± 10%, 12:12-h light: dark cycle (lights on, 0700 to 1900) with a light intensity of 300 lx at 1 m above the floor, and were allocated to four groups by a two-stage randomization: control group (*n* = 32, 20 for histological comparison to the CRS model and 12 for MSC administration and histological control to the MSC model) and an Af exposure group (*n* = 32, 20 for histological comparison to the 1st control group and 12 for MSC administration, to the CRS/MCS model); for the first stage we selected a control group (*n* = 20) and an Af exposure group (*n* = 20); for the second stage the CRS with the MSC exposure group (*n* = 12) and the second control group with MSC administration (*n* = 12). The second part of our study was a continuation of the first part, mentioning that in the MSC exposure group, there were 12 CRS mice exposed to Af for 12 weeks and the second control group was composed of 12 mice with 12 weeks of intranasal administration of saline solution; the 24 animals were not euthanized in the first stage of the study. All protocols were approved by the Ethics Committee of the University (Authorization No 73/20 Feb 2014) and were conducted in accordance the EU Directive 63/2010. All the animals were quarantined before the onset of the study. For environmental enrichment, autoclaved braided cotton dental rolls were used (Celluron, Hartmann, Germany). The present study was designed and performed in accordance with the “ARRIVE Guidelines for Reporting Animal Research” [6].

#### 2.1.2. Procedure and Data Collection

##### CRS Mouse Model

The mycelia extract of Af used for the intraperitoneal sensitization and for intranasal applications was obtained from the Faculty of Veterinary Medicine Cluj-Napoca. The first step was the sensitization of the exposure group by a single intraperitoneal injection of Af 200 µg absorbed on 2 mg alum in 0.5 mL of phosphate-buffered saline solution (PBS) [7]. The control group received PBS injected according to the same protocol.

One week after sensitization, the exposure group received the first dose of 10 µg (5 µL) of Af antigen bilaterally in the nasal cavities, through a micropipette, in a supine position, after general anesthesia with isoflurane (Aerrane, Baxter, UK) using a standard vaporizer (EZ-Anesthesia, Sarasota, FL, USA). The control group received 5 µL of normal saline solution in both nostrils. This operation was repeated in both groups 3 days per week for 12 consecutive weeks, according to Khalid et al.’s murine model protocol, for CRS [8].

##### MSC Isolation and Culture

Adipose tissue samples were harvested from the subcutaneous adipose tissue of female CD1 mice (*n* = 6). The samples were washed with PBS and transferred into transport medium DMEM (Sigma-Aldrich, Saint Louis, MO, USA) supplemented with 10% fetal calf serum (Hyclone) and 1% Antibiotic-Antimycotic 100×(Gibco). Cells were isolated using a mixed enzymatic-explant method. The cell suspension and the explants were cultured in a DMEM/F12 propagation medium (Sigma-Aldrich, Saint Louis, MO, USA) supplemented with 10% fetal calf serum (Hyclone) and 1% Antibiotic-Antimycotic 100×(Gibco) in standard culture conditions. After 6 passages, the isolated cells were assessed for surface markers (CD44, CD105) [9,10].

##### MSC Allograft in the CRS Mouse Model

The delivery mechanism, which assures an optimal distribution of MSCs, is extremely important. The direct administration of MSCs through topical or direct injection is currently most used. Cho KS et al. demonstrated in 2009 the MSCs’ immunomodulatory effect on CRS mouse model injected via the mouse tail vein [5]. Thus, it can be inferred that MSCs have the potential to localize the damaged organs.

The second step of the study consists in the inoculation of MSCs via the ophthalmic vein. The aim of this stage is to evaluate whether MSCs have a favorable influence on the evolution of CRS and also the homing ability of the cells used. After the general anesthesia, 1 × 10^6^ characterized MSCs in 100 µL PBS were injected intravenously using the retro-orbital injection method described by Yardeni et al. to each remaining animal after the first stage (both in the experimental and the control group) [10].

##### Histological Analysis–CRS Mouse Model

A total of 40 animals (20 Af exposed and 20 from the control group) was euthanized after 2, 4, 8 and 12 weeks of the 1st stage of study. Euthanasia was carried out using isoflurane, for the fixation of the animals’ heads formaldehyde 10% was used for 1 day, the skulls were trimmed and kept in nitric acid 5% for 5–6 days, at 4 °C, for decalcification. We created four coronal sections according to the standard levels: I caudal imaginary line to the incisors, II an imaginary line to halfway of I to III, III an imaginary line to anterior margin to orbit and IV posterior margin to orbit. The sections obtained were fixed in paraffin and were stained using the Hematoxylin and Eosine (H&E) method. Finally, the histological sections were examined under an Olympus BX41 light microscope and images were obtained with an Olympus UC30 camera. The inflammation was evaluated by the severity or grade of inflammation (Table 1). All sections were examined by a blinded examiner.

##### Histological Analysis after MSC Exposure

After MSC administration, 3 (*n* = 3) mice of each group were euthanized on days 4, 5, 6 and 7 using isoflurane inhalation. The nasal mucosa was prepared exactly as in the first stage. The main purpose was to determine the stem cells in the nasal mucosa, and the second purpose was to analyze the influence of MSCs in the CRS treatment. The standard inflammatory markers were evaluated and compared with those of the control group and of the Af exposure group, also, the stem cells were counted and compared with those in the control group, and the evolution of inflammation after MSCs administration was evaluated. A comparison was made between the first stage of study on Af exposure group and the second study stage on the CRS induced group.

##### Confocal Scanning Laser Microscopy (CSLM) Analysis

For the fluorescent detection of stem cells, the trimmed tissues were briefly washed with PBS, embedded in Optimal cutting temperature media (OCT), sectioned at 20 µm using a Leica CM 1850 cryostat and collected on poly-l-lysine-coated glass slides. The nuclei were stained with Blue pseudocolor (DRAQ5^®^), slides were mounted with an antifade-aqueous medium (Mowiol 4-88-Carl Roth) and stored in dark until analysis.

Samples were analyzed using a Zeiss LSM 710 Confocal Laser Scanning unit (equipped with Ar and HeNe lasers) mounted on an Axio Observer Z1 Inverted Microscope. Images were recorded using a Plan Apochromat 63 × oil immersion objective and further animalized and processed using the standard Zeiss ZEN software [9].

##### Statistical Analysis

Results were analyzed using SPSS Statistics version 19.00 for Windows (IBM). Ordinal data was expressed as median and 25%–75% (non-normal distribution). Nominal data was characterized by frequency and percentage. Statistical analysis for the CRS mouse model: histological analysis was performed using the Mann-Whitney (comparison between two groups), Kruskal-Wallis (comparison between more than two groups), Friedman test or chi-square test, whenever appropriate. Statistical analysis for the CRS evolution after the MSC administration was performed using the Mann-Whitney (comparison between two groups), Kruskal-Wallis (comparison between more than two groups) Friedman test or chi-square test, whenever appropriate. In all the analyses, p-values were evaluated for the 5% and 10% significance values, according to the probabilities obtained.

## 3. Results

### 3.1. CRS Mouse Model: Histological Analysis

**Figure 1:**
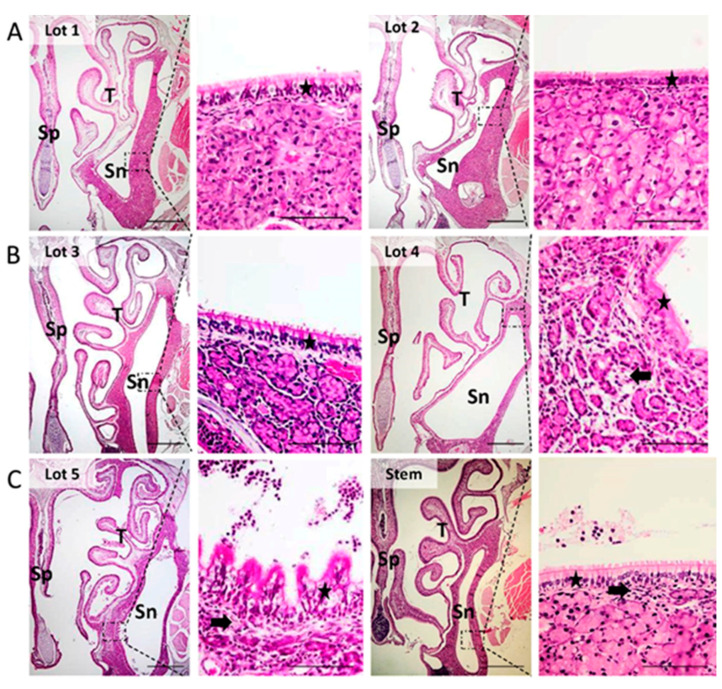
Histological analyses. (**A**) Lot 1. Control group. Lot 2 CRS mouse model after 2 weeks of Af exposure. (**B**) Lot 3. CRS mouse model after 4 weeks of Af exposure. Lot 4 CRS mouse model after 8 weeks of Af exposure. (**C**) Lot 5 CRS mouse model after 12 weeks of Af exposure. Stem: MSC mouse model-4 days after MSCs i.v. administration. Sp = septum, Sn = sinus, T = turbinate. The black star mark epithelial inflammatory modifications (hyperplasia, squamous metaplasia, Goblet cells–Lot 5) and the black arrow mark the presence of inflammatory cells infiltrate (mononuclear cells).

The microscopic examination of rhinosinusal mucosa showed significant differences between the Af exposure group and the control group at the end of 12 weeks of the Af exposure period. After 2 weeks of nasal Af exposure, the histological changes are as follows: subepithelial fibrosis, diffuse shortage of epithelial cilia, focal areas of respiratory epithelial hyperplasia and accumulation of proteinaceous material, rich in cell debris, which compress the adjacent structures (sinus, especially at the level of the medial wall) (Figure 1, Lot 2).

8 weeks after the onset of Af exposure, there is a cystic distension of the vomeronasal organ and paranasal sinus by a mucopurulent exudate (catarrh), associated with focal mucosal desquamation, glandular atrophy, Goblet cell hyperplasia (*p* = 0.005), diffuse infiltration of the submucosa with neutrophils and osteochondral lysis (*p* = 0.006). There is also squamous metaplasia of the respiratory mucosa covering the nasal septum and atrophy of the nasal turbinates associated with focal mucosal hyperplasia, diffuse edema of the lamina propria and fibrosis (with secondary atrophy of the seromucous glands) (Figure 1, Lot 4). At the end of first part of our study, respectively after 12 weeks of Af exposure, the inflammation and secondary tissue destruction is marked. The sinus cavities are distended by a mucopurulent exudate rich in cell debris, with an extensive osteolysis of the maxillary bones and the nasal meatus is obstructed by an abundant exudate as described above for sinuses. The nasal septum presented focal areas of chondrolysis. The mononuclear cell infiltrate was diffuse and classified as severe with a clear statistical significance (*p* = 0.005); there was also goblet cell hyperplasia (*p* = 0.004) (Figure 1 Lot 5, Table 2 and Table 3).

If the subepithelial fibrosis, a focal subepithelial edema, discrete mononuclear cell infiltrate and shortened cilia were present after 4 weeks of Af exposure, at the end of the Af exposure, all these parameters indicated a severe diffuse chronic inflammation, associated with a diffuse severe hyperplasia and a mature diffuse squamous metaplasia. Statistical analysis confirms this progressive evolution (Table 2 and Table 3).

The eosinophillic cells’ presence in the first stage of the study had an interesting evolution, with a real apparition after 4 weeks of Af exposure and a constant presence (grade 2-moderate inflammation) at week 8 and 12, associate with hyaline globules and Charcot-Leyden crystals (Table 2 and Table 3).

A thin layer of the catarrhal exudate (consisting of variable numbers of neutrophils admixed with mucus and few necrotic cell debris) expanded caudally, covering partially the olfactory epithelium. Occasionally, in all the examined experimental groups, a few intracytoplasmatic eosinophilic globules (hyaline droplets) were noticed within both respiratory and olfactory epithelium. As previously described by Renne et al. (2009) and Ramos et al. (2018), eosinophilic globules were considered to be incidental background change. No other changes were noted with the olfactory epithelium in any of the experimental groups [13,14].

### 3.2. Detection of MSC in Nasal Mucosa

Because the CD 44 is the marker identified immediately after the MSC adhere to an organ or tissue [15], the histological examination must be performed in the first seven days after MSC administration.

Digital image acquisition was performed using a triple filter for red, green and blue spectra. We chose Alexa 546 spectra for our stem cells marker. Fluorescent signals for CD44 were located especially in the cell membrane.

We analyzed four specimens for each euthanized mouse and for the final results; we used an average of MSC number per mouse and per day.

**Figure 2:**
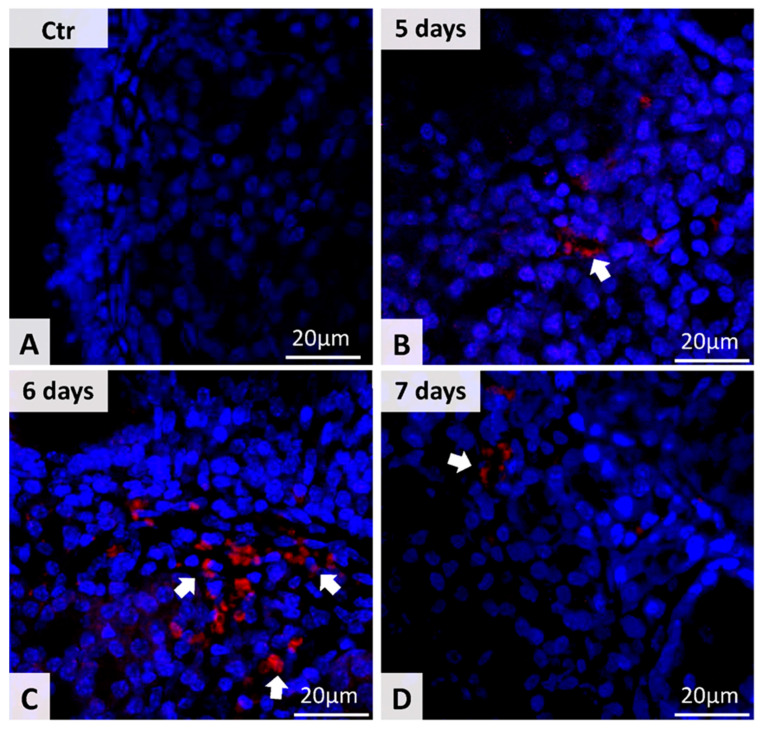
MSC detection in CSLM analysis (**A**) Control group (**B**) 5 days after MSCs administration on the CRS mouse model. (**C**) 6 days after MSCs administration on the CRS mouse model. (**D**) 7 days after MSCs administration on the CRS mouse model. White arrow indicates the Fluorescent signals for CD44.

**Figure 3:**
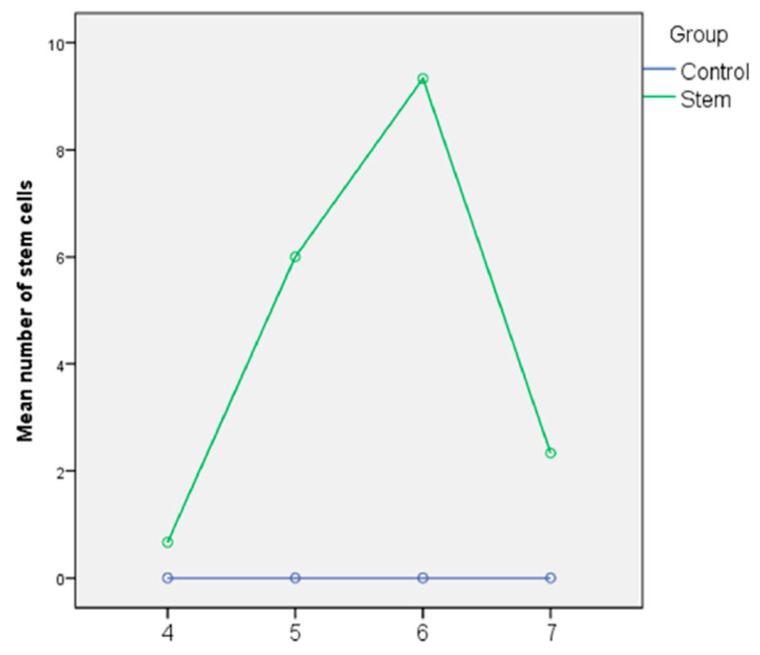
Number of MSCs evolution (CRS mouse model daily distribution)-Comparison with the control group.

On day 4 after the MSC administration, only mice with induced CRS presented MSCs in the nasal mucosa (2 cells), identified in two corresponding four samples. The control subjects (mice without CRS but with MSC injected) did not present MSCs in the nasal mucosa (Figure 2 and Figure 3, Table 4).

On day 5, the number of MSC identified in the nasal mucosa of euthanized mice in the second experimental group was higher than in the previous days. The control subjects did not present MSC in the nasal mucosa (Figure 2 and Figure 3, Table 4).

On day 6, we recorded a peak in the number of MSCs identified in the nasal mucosa of experimental subjects, mentioning that the control subjects still present no MSC (Figure 2 and Figure 3).

On day 7, the number of MSCs detected in the nasal mucosa of experimental subjects is in regression (p = 0.033), the control subjects are free from MSC in the nasal mucosa (Figure 3, Table 4).

### 3.3. CRS Evolution after the MSC Administration

On day 4, in the case of the MSC subject detected by fluorescence microscopy, the histological analysis indicated a sinus empyema accompanied by a mainly neutrophilic infiltrate (also, few macrophages are present) of the submucosa, focal osteolysis, subepithelial fibrosis mucosal hyperplasia (focally polypoid-shaped), moderate eosinophilic infiltrate, but a strong Charcot-Leyden crystals presence. The septal and turbinate epithelium also presented areas of ciliary loss and squamous metaplasia. All these histological changes indicate a severe chronic inflammation. The other two subjects presented a moderate degree of inflammation (Figure 1, Table 5).

On day 5, the mononuclear cell infiltrate is also abundant in the transmural wall (*p* = 0.043). Within the sinus mucosa, there are focal areas of ulceration alternating with focal areas of mucosal hyperplasia or mucosal metaplasia (Table 5). The cilia are diffusely absent. The nasal septum and nasal turbinate’s also present the above mentioned histological changes, associated with osseocartilaginous lysis. No significant inflammation was noticed for the control group.

On day 6 after the MSC infiltration, the predominant histological changes are at the level of the glandular epithelium, which is atrophied, the mononuclear cell infiltrate is in regression (*p* = 0.068), also the eosinophilic cells are in reduced number, but many Charcot-Leyden crystals are still present. The sinus catarrh is absent and the sinus space is distended. The fibrosis of the submucosa is diffuse (present for both sinuses, septal and turbinate mucosa) (Table 5). The epithelial hyperplasia and squamous metaplasia, although present, concern smaller areas of the epithelium. The ciliated cells are present, but cilia are shortened.

On day 7, the histological lesions are minimal to mild, consisting of diffuse, mild fibrosis of the submucosa with compression and atrophy of the seromucous glands and a mild submucosal mononuclear infiltration; the mononuclear cell infiltrated (*p* = 0.114), the number of Goblet cells is significantly reduced (*p* = 0.114), the eosinophilic cells denote a minimal grade of inflammation (*p* = 0.003). Few areas of shortened of cilia, epithelial hyperplasia and squamous metaplasia were found. Most of the sinus and nasal mucosa are normal.

## 4. Discussion

The nasal epithelium plays an essential role in mediating the contact of host tissue with environmental factors. The absence of the barrier function of the nasal epithelium is the prerequisite for the development of inflammation and infection [16]. Nasal epithelium remodeling after physical injuries includes epithelial hyperplasia, loss or shortening of cilia, edema, goblet-cell hyperplasia or metaplasia and basement membrane denudation [14]. The upper airway epithelium’s response to external aggression is a very well-coordinated process, facilitated by eosinophils and several molecules like cytokines, lipid mediators, growth factors and transcription factors. These mediators control complex processes such as migration, proliferation and cell differentiation [17,18].

According to many studies, CRS is defined as a persistent inflammation of nasal and paranasal mucosa for a minimum of 12 weeks. The European Position Paper on Rhinosinusitis and Nasal Polyps 2020 establishes a basic classification of CRS: chronic rhinosinusitis without nasal polyps (CRSsNP) and chronic rhinosinusitis with nasal polyps (CRSwNP) [18]. Likewise Luo Ba et al. demonstrated in a recent article that the inflammatory profile for CRSwNP could be classified as atopic and nonatopic with a real differentiation in what concerns the inflammatory response [19].

CRS has an important socioeconomic impact in terms of quality of life and costs for the therapeutic strategies. If the CRS current medical treatment is effective in many cases, a substantial number of patients continue to have persistent nasosinusal symptoms and a significant number of these are considered to be candidates for surgical treatment, usually ESS [3]. The goals of ESS are to reestablish sinus drainage and to create an optimal space for an efficient application of topical drugs [20,21]. However, ESS could entail complications such as postoperative synechia or restenosis of the maxillary antrostomy, with an important incidence of revision sinus surgery [4,22]. These aspects prompt a new direction in the research of the CRS treatment.

The characteristic feature for CRS is the chronic inflammation of the nasal mucosa, the final result of which involves significant inflammatory changes: (a) imbalances in the interaction between hosts and external aggressive factors, (b) the host inflammatory parameters with an unpredictable evolution like the inflammatory cells infiltrate [23,24,25,26], cilia disappearance, fibrosis, edema, hyperplasia of secretory cells, disruption of epithelium, (c) abnormal remodeling starting with the basement membrane and finishing with the polypoid aspects of epithelium, (d) alteration of the regulation mechanism of Th1, Th2 and IG2 [6], eicosanoid pathway [3,16,17,18,19].

In many experimental studies, Af is involved in the pathophysiology of allergic fungal chronic sinusitis or allergic rhinitis, but also in human upper airway diseases [4,27,28]. The nasal mucosa examined in our study showed lesions characteristic for Af pathogenesis, such as bone or cartilaginous retraction (nasal septum or sinus wall), which is explained by Yan et al. as the direct intrusion of hyphal masses [29]. Our histopathological findings highlight the inflammatory and chronic character of the lesions represented by epithelial changes, and also explain the allergic character by the eosinophilic infiltration and the Goblet cells proliferation.

Currently, surgery remains the principal treatment of CRS unresponsive to maximal medical therapy. [30].

MSCs represent an option for the management of many diseases based mainly on the fact that MSCs can identify the injured organs, but also to their capacity to differentiate in vitro into several types of cells [31].

MSCs display anti-inflammatory and immunomodulatory characteristics [32,33]. Animal experiments suggest that MSCs could have a potential therapeutic contribution in the treatment of chronic obstructive pulmonary disease (COPD) [33]. Recently, it was demonstrated that MSCs have immunomodulatory effects in nasal polyposis [34,35]. Adipose tissue-derived MSCs reduced the proportions of activated CD4+ and CD8+ T cells and Th2 immune response. Bone marrow-derived MSCs repressed the CD4+ and CD8+ T cell production, increased the prevalence of regulatory T cells and transformed the cytokine profile from an inflammatory to an anti-inflammatory pattern. These results suggest that MSCs are able to modulate the inflammatory process in CRSwNP and hold perspective of future therapy of this disease.

Recent technological advancements have enabled the delivery of targeted and effective treatments in CRS cases. Thus, precision medicine [36] in CRS involves ordering patients into subgroups (phenotypes and endotypes) and delivering customized treatment plans. Currently, new treatment options are suggested in CRS cases: monoclonal antibodies, targets against sialic acid-binding Ig-like lectin 8 and thymic stromal lipoprotein (anti-Siglec-8 and anti-TSLP), PGD2 receptors antagonists. Undoubtedly, MSCs present a potential avenue of research in the individualized CRS treatment. The association of allergic rhinitis with CRS is very well established. Allergic rhinitis as a co morbidity situation to CRS may lead to new treatment policies in the future, with the potential role of MSCs, in cases of CRS with nasal polyps. Based on the theory of united airways, which considers respiratory tract as a unified tract, further clinical applications may be advantaged. The cases of uncontrolled CRS, that represents 30%–40% of CRS cases is an additional interesting issue.

The delivery mechanism is not standardized, but it is most important because the purpose is to obtain a high number of MSC in the injured area. Accessible peripheral areas, such as osteoarticulations, tendons or muscles of the limbs, corneal or subretinal space and skin, allow local administration via injection with or without ultrasound guidance or topical application, both methods having demonstrated a good efficiency [37,38,39,40,41]. For the other localizations, like abdominal organs, bones or central nervous system, the MSC direct administration is difficult and the intravenous MSC (i.v.) administration is efficient and easy. Fang et al. showed in 2007 that the i.v. MSCs can be used with good results in the detection and reparation of the damaged liver [42]. To optimize the chances that the MSCs concentrate in the nasal mucosa, we chose ophthalmic i.v. administration on the juxta-lateral side. The histopathological results indicate a high number of MSCs 5 or 6 days after the administration in the MSC experimental group. In the control group, no MSCs have been identified by histopathological methods, which highlights that the damaged tissue signals through specific mediators or receptors and facilitate the migration, adhesion and infiltration of MSCs in the injured area [31]. The mechanism of MSC inflammation modulation is not clearly yet understood but its existence is demonstrated by the capacity of MSCs to repair the damaged tissues and to reestablish the normal functions of the respective organs [43]. Linda L Black et al. started from the idea that MSCs secret Interleukin 1 (Il-1) and receptor antagonist (Il-1ra), which determined the inflammation and fibrosis and lead to a functional improvement in the animals treated with MSCs [35]. Yang et al. showed the immunomodulatory role of MSCs in cases of allergic rhinitis in mice, where IgE, Ig G_2a_ levels were higher in the MSCs exposure group than in the control group and the final result was a reduction of allergic inflammation [44]. In the present study, the inflammation was quantified using histopathological inflammatory parameters. Immediately after the MSCs administration, the inflammatory parameters persisted (day 4), the mononuclear cells were still abundant, the cilia were absent from large areas, fibrosis, epithelial hyperplasia and squamous hyperplasia were at the same level as at the end of first part of study, considering that the MSCs were poorly evidenced in the injured mucosa. When the number of MSCs increased in the pathological mucosa (days 5 and 6), the inflammation regressed and the number of mononuclear cells decreased, the cilia were shortened or normal, the number of Goblet cells and eosinophilic cells was reduced and the fibrosis, epithelial hyperplasia and squamous metaplasia were statistically improved.

Our study had some limitations, which could be mitigated in subsequent studies. First, the histological analysis focuses on the respiratory mucosa, the olfactory changes are not described and highlighted in detail. Imaging analysis with micro- CT or MRI could also support and show more obvious changes of the mucosa in CRS. A new mouse CRSwNP model study, with the evaluation of the clinical evolution and the imaging analysis could represent a prospective work.

## 5. Conclusions

This study opens a new perspective for CRS management. Considering this is the first pilot study for MSC administration on the CRS mouse model, further research is needed for confirmation of this therapeutic strategy.

## Figures and Tables

**Table 1:** Parameters used in the histological assessment [11,12].

Inflammatory Markers	Grade	Description
Mononuclear (inflammatory) cell infiltrate	0	Normal aspect
		0–10 cells/HPF (high-pass filter, 40×)
	1	Discrete inflammation
		11–30 cells/HPF
	2	Moderate inflammation
		31–50 cells/HPF
	3	Severe inflammation
		>50 cells/ HPF
Goblet cells	0	Normal aspect
		0–10 cells/HPF (high-pass filter, 40×)
	1	Discrete inflammation
		11–30 cells/HPF
	2	Moderate inflammation
		31–50 cells/HPF
	3	Severe inflammation
		>50 cells/ HPF
Edema	0	No edema
	1	Focal subepithelial edema
	2	Diffuse subepithelial edema
	3	Diffuse subepithelial and intraglandular edema
Cilia	0	Normal aspect
	1	Shortened cilia
	2	Dotted cilia disappearance
	3	Lack of cilia
Fibrosis	0	No fibrosis
	1	Subepithelial fibrosis
	2	Subepithelial and interglandular fibrosis
	3	Diffuse fibrosis (subepithelial and interglandular) with compression atrophy of glands and capillaries
Epithelial hyperplasia	0	No epithelial hyperplasia
	1	Dotted hyperplasia
	2	Diffuse hyperplasia
Squamous metaplasia	0	No squamous metaplasia
	1	Immature squamous metaplasia
	2	Mature squamous metaplasia/diffuse
Eosinophilic cells	0	Normal aspect
		0–5 cells/HPF (high-pass filter, 40×)
	1	Discrete inflammation
		6–10 cells/HPF
	2	Moderate inflammation
		11–15 cells/HPF
	3	Severe inflammation
		>15 cells/HPF

**Table 2:** Statistical analysis of the inflammation parameters evolution on the CRS mouse model.

Variable		Week 2	Week 4	Week 8	Week 12	*p*
Mononuclear cell infiltrate *		0 (0; 0.5)	1 (0.5; 1.5)	3 (2; 3)	3 (2.5; 3)	0.001
Fibrosis *		1 (0; 1)	1 (1; 1.5)	3 (3; 3)	3 (2.5; 3)	0.001
Edema *		0 (0; 0)	0 (0; 1)	3 (3; 3)	3 (2; 3)	0.001
Cilia *		1 (1; 1)	1 (1; 1)	2 (1.5; 2.5)	2 (2.5; 2)	0.002
Goblet cells *		0 (0; 0)	0 (0; 0)	2 (1.5; 3)	2 (2; 2.5)	0.001
Eosinophilic cells		0 (0; 0)	1 (1; 1)	2 (1; 2)	2 (2; 2)	0.001
Epithelial Hyperplasia **	Grad 0	5 (100%)	5 (100%)	-	-	<0.001
	Grad 1	-		5 (100%)	5 (100%)	
Squamous metaplasia **	Grad 0	5 (100%)	5 (100%)	-	-	<0.001
	Grad 1	-		5 (100%)	5 (100%)	

* expressed as median and 25–75%; ** expressed as frequency and percentage.

**Table 3:** Statistical comparison between CRS model and control group.

Variable		Control Group	Week 8	Week 12
Mononuclear cell infiltrate *		0 (0; 0.5)	3 (2; 3)	3 (2.5; 3) ^++^
Fibrosis *		0 (0; 0)	3 (3; 3) ^+^	3 (2.5; 3) ^+^
Edema *		0 (0; 0)	3 (3; 3) ^+^	3 (2.5; 3) ^+^
Cilia *		0 (0; 0)	2 (1.5; 2.5) ^+^	2 (2.5; 2) ^+^
Goblet cells *		0 (0; 0)	2 (1.5; 3) ^+^	2 (2; 2.5) ^+^
Eosinophilic cells		0 (0; 0)	2 (1; 2)	2 (2; 2)
Epithelial Hyperplasia **	Grad 0	5 (100%)	- ^+^	- ^++^
	Grad 1	-	5 (100%) ^+^	5 (100%) ^++^
Squamous metaplasia**	Grad 0	5 (100%)	-	-
	Grad 1	-	5 (100%) ^+^	5 (100%) ^++^

* expressed as median and 25–75%; ** expressed as frequency and percentage; ^+^ statistically significant difference between martor and group 4; ^++^ statistically significant difference between martor and group 5.

**Table 4:** MSC Group Statistics.

	Group	N	Mean	Std. Deviation	Std. Error Mean
Number of stem cells	Control	12	0.00	0.000	0.000
	Stem	12	4.58	3.942	1.138

**Table 5:** Daily evolution after MSC administration–statistical analysis.

Variable		Day 4	Day 5	Day 6	Day 7
Mononuclear cell infiltrate *		3 (3; 3)	3 (2; -)	2 (1; -)	1 (1; 1)
Fibrosis **	2	-	-	-	1 (33.3%)
	3	3 (100%)	3 (100%)	3 (100%)	2 (66.7%)
Edema *		3 (2; -)	1 (1; -)	1 (1; 1)	0 (0; -)
Cilia *		3 (3; 3)	2 (1; -)	1 (1; 1)	0 (0; -)
Goblet cells *		3 (3; 3)	2 (2; -)	1 (1; -)	1 (0; -)
Eosinophilic cells		2 (2; 2)	1 (1; 1)	1 (1; 1)	1 (0; 1)
Epithelial Hyperplasia **	Grad 0	-	-	-	-
	Grad 1	3 (100%)	3 (100%)	3 (100%)	3 (100%)
Squamous metaplasia **	Grad 0	-	-	-	-
	Grad 1	3 (100%)	3 (100%)	3 (100%)	3 (100%)

* expressed as median and 25–75%; ** expressed as frequency and percentage.
